# How do low-income single-mothers get by when unemployment strikes: Patterns of multiple program participation after transition from employment to unemployment

**DOI:** 10.1371/journal.pone.0274799

**Published:** 2022-09-22

**Authors:** Chi-Fang Wu, Yu-Ling Chang, Soohyun Yoon, Salma Musaad

**Affiliations:** 1 School of Social Work, University of Illinois at Urbana–Champaign, Urbana, Illinois, United States of America; 2 School of Social Welfare, University of California, Berkeley, California, United States of America; 3 Department of Pediatrics, USDA/ARS Children’s Nutrition Research Center, Baylor College of Medicine, Houston, Texas, United States of America; FAME|GRAPE, POLAND

## Abstract

Little is known about longitudinal patterns of welfare program participation among single mothers after they transition from employment to unemployment. To better understand how utilization patterns of these welfare programs may change during the 12 months after a job loss, we used the 2008 Survey of Income and Program Participation to examine the patterns of participation in Medicaid, the Supplemental Nutrition Assistance Program, Temporary Assistance for Needy Families, and unemployment insurance among 342 single mothers who transitioned from employment to unemployment during the Great Recession. Using sequence analysis and cluster analysis, this paper identified four distinct patterns of program participation: (a) constantly receiving in-kind benefits; (b) primarily but not solely receiving food stamps; (c) inconsistent unemployment insurance or Medicaid-based benefits; and (d) limited or no benefits. Almost two-fifths of our sample of single mothers received inconsistent, limited, or no benefits. Results of the multinomial regression revealed that race, work disability, poverty, homeownership, and region of residence were significant factors that influenced whether study subjects participated in or had access to social safety net programs. Our findings illustrate the heterogeneity in patterns of multiple program participation among single mothers transitioning from employment to unemployment. Better understanding these varied patterns may inform decisions that increase the accessibility of US social safety net programs for single mothers during periods of personal economic hardship.

## Introduction

Since the onset of the COVID-19 pandemic in March 2020, single mothers have been at high risk of job loss and have struggled to find new employment and maintain economic security [1]. This is partly attributable to the pandemic’s disproportionate effect on retail and service industries [2], sectors which employ many single mothers [3]. The unemployment rate for single mothers increased from 5.9% in 2019 to 10.4% in 2020 [4]. Additionally, the official poverty rate of families led by single mothers was 32.1% in 2020, more than double that of households led by single fathers and more than five times that of married-couple families with children [5]. Beyond unemployment, COVID-19 also prompted widespread school and childcare center closures, placing an enormous childcare burden on families. Married mothers left the workforce at unprecedented rates [6,7], but many single mothers lacked the economic support necessary to have this option. Single mothers seeking childcare also faced disease risk, making it difficult to depend on informal care provided by grandparents, relatives, or neighbors [6,8]. Given the far-reaching impact of COVID-19 on unemployment and its related effects on low-income single-mother families, it is crucial to better understand the ways in which this group utilizes social safety net programs following job loss and income reduction.

Evidence shows that many single mothers experiencing poverty (e.g., [9])—particularly those in households in which the single mother is unemployed [10]—received support from multiple safety net programs. However, little is known about the sequential patterns of multiple program participation among low-income, single-mother families after these mothers become unemployed. Understanding the patterns in sequences and combinations of enrollment in different programs, and their associated sociodemographic characteristics, can inform policies and practices to improve access to social safety programs for single mothers who experience unemployment. Without these nuanced understandings, policymakers and providers lack the data needed to optimize the enrollment in and positive effects of these programs for this generally high-need, at-risk population.

To address this knowledge gap, the current study used data from a nationally representative panel sample from the 2008 Survey of Income and Program Participation (SIPP) to examine program participation by single mothers after job loss. We conducted sequence and cluster analyses to examine the type, order, and duration of multiple program participation by low-income single mothers transitioning from employment to unemployment. Data from the 2020 SIPP panel that covers the period of the pandemic recession is not yet available. Consequently, the 2008 panel offers the best data for studying longitudinal patterns of multiple program participation during an economic recession during which the federal government temporarily expanded social safety net programs under the American Recovery and Reinvestment Act of 2009 [11,12]. Because the conditions reflected in the 2008 panel are similar to expansions temporarily implemented to mitigate the impacts of COVID-19, we believe our analyses offer timely insights relevant to the current policy and support service landscape.

The 2008 SIPP data used in this study captures the impact of the Great Recession, which significantly impacted single mothers [13,14]. A growing body of evidence indicates that the Great Recession had a major negative economic impact on single mothers [15,16].

Unemployment rates for single mothers increased substantially during that time, as did their participation in the Supplemental Nutrition Assistance Program (SNAP) [13,14] and an array of other social safety net programs [10,11,13,17–21].

The three aims of the current study of single mothers transitioning from employment to unemployment were as follows: (1) examine longitudinal patterns of multiple program participation within the 12 months following unemployment; (2) identify multiple program participation cluster groups; and (3) analyze characteristics associated with each multiple program participation cluster group. The results reveal the combination of in-kind and cash programs used by struggling families in a period of recession, illustrating patterns of multiple program participation not previously identified. Specifically, we illuminate the timing and patterns of complex multiple program participation among single mothers following job losses. Understanding how unemployed single mothers used social safety net programs during the Great Recession is a key prerequisite to creating and revising policies and programs designed to address under- and unemployment for this demographic. Once the patterns of multiple program use are identified, they can be used to study outcomes: namely, the efficacy of these varying patterns in enabling single mothers to overcome potential economic hardships. Findings derived from the 2008 SIPP data reported in this study provide an empirical foundation for guiding policy as the federal government considers whether to extend the access to the unemployment insurance (UI) system deployed in the COVID relief bills and whether to adopt policies that provide support for all families with young children [22].

## Literature review

Evidence suggests that participation in multiple safety net programs is critical because enrollment in a single support program is often insufficient to address the competing needs of participating families [23]. Single mothers are at high risk of experiencing material hardship and nutritional and mental health issues, and their children are at high risk of poor academic performance and social-behavioral problems (e.g., [24–26]). Unsurprisingly, many single mothers qualify for more than one social safety net program, reflecting a broad pattern in which people who qualify for one program often qualify for—and participate in—multiple programs (e.g., 99% of families receiving Temporary Assistance for Needy Families [TANF] and 89% of families receiving SNAP also participate in Medicaid/Children’s Health Insurance Program [27]).

The broad changes to key safety net programs brought by the 1996 welfare reform have intensified the need to examine multiple program enrollment by single mothers. TANF has become less responsive to economic needs and is increasingly unavailable to many families at the bottom of the income distribution [28,29]. Similarly, many people who work in part-time jobs, in non-traditional jobs, or in jobs with volatile work schedules do not qualify for UI benefits. While SNAP has remained a critical pillar of support for low-income families, especially those experiencing unemployment and underemployment [20,28], TANF fills a smaller percentage of the poverty gap and has been less responsive to the needs of low-income families than other safety net programs [30]. Post-reform changes may render related data collected prior to 1996 obsolete, preventing interventionists and policymakers from making data-driven decisions about service use and program participation. For this reason, it is critical to track single mothers’ current engagement with the safety net system to inform policymaking and, ultimately, provide low-income single mothers with a path out of poverty.

### Multiple program participation patterns

Although empirical studies have defined national trends in program participation rates for a variety of social safety net programs (e.g., [11,20]), comparatively limited research has focused on enrollment patterns among low-income single-mother families, despite their established vulnerabilities. For instance, using SIPP data, one study [19] tracked the rate and length of participation of low-income single mothers in six programs from 2004 to 2008. Over that time period, researchers observed substantial increases in participation in all programs except TANF. Similarly, there were slight increases in average enrollment duration for all programs except Supplemental Security Income (SSI). While this program-by-program analytic approach provided an overview of program participation and cumulative benefit, questions about person-centered, longitudinal experiences of multiple program participation remain unanswered. Additionally, Cancian and colleagues [31] found that once single mothers started receiving benefits, participation in three programs (i.e., cash assistance, SNAP, and Medicaid) was the most common pattern. Over time, the percentage of single mothers participating in both SNAP and Medicaid without receiving cash assistance increased more than the percentage of single mothers participating in all three programs [31]. However, their research focused exclusively on mothers who entered Wisconsin’s TANF program (W-2) and did not consider the impact of employment status over time.

Four studies have addressed patterns of multiple program participation among single-parent families during or after the Great Recession (2008–2013). Moffitt [32] found that about one in three single-parent families receiving SNAP in 2008 also benefited from at least one other program (e.g., TANF, Medicaid, housing benefits, UI, or SNAP for Women, Infants, and Children [WIC]). Seefeldt and colleagues [9] found that TANF- and UI-receiving families showed the highest percentage of participation in both SNAP and public health insurance. Similarly, Cancian and colleagues [33] found that TANF-receiving families tend to participate in a greater number of programs (e.g., SNAP, Medicaid, SSI, and UI) than SNAP-receiving families. For example, about half of SNAP families participated in one or two programs, while more than 95 percent of TANF families participated in three or more programs. Given that about 90 percent of TANF families and two-thirds of SNAP families were headed by single mothers in 2010, these results underline the significance of understanding multiple program participation among single mothers. These three studies showed that in-kind benefits, such as Medicaid or SNAP, played significant roles in supporting single-parent families during this period of economic crisis.

Finally, Chang and Wu [17] studied monthly program participation in seven programs (i.e., UI, SSI, TANF, SNAP, Medicaid, housing assistance, and childcare subsidies) from 2008 to 2013. Specifically, they analyzed patterns of seven types of monthly program participation by employment trajectory group. They found that low-income single mothers experiencing unstable employment trajectories were more likely to receive in-kind basic needs benefits than family benefits packages that included cash assistance. However, their study did not examine sequences of multiple program participation in relation to the timing of unemployment, and thus they could not identify individual patterns of benefit packages used by single mothers in the months after they lost their jobs. The current study addresses the resulting gap by tracing the dynamic post-unemployment patterns of multiple program participation and unemployment, which were not clearly explained in previous studies. It examines the order and timing of low-income single mothers’ receipt of UI and other social assistance benefits following job loss, a time of great need for cash benefits due to reduced earnings, which gives this study significant analytical advantages over past research. Unlike previous studies that only examined combinations of benefit packages at a single point, the current study analyzes how low-income single mothers used different welfare benefits sequentially during a time of high economic instability.

### Multiple program participation sequences

Few studies of multiple program participation have examined sequences of program participation or longitudinal changes in program participation over time. One study [34] used sequence analysis to examine welfare participation patterns among families in which a child had been removed by child protective services. Although that study focused on child removal rather than employment transition, it concluded that the low-income families had only limited and tenuous options for cash assistance programs and identified six clusters (i.e., patterns) of monthly benefit participation: short spells of TANF, lose TANF, gain TANF, steady TANF, steady SSI, and TANF+SSI.

Two other studies used cluster analysis to examine how low-income families with young children engage with benefits packages [35,36]. Both studies identified four distinct cluster groups based on employment status and benefit receipt and found that these cluster groups were associated with specific demographic characteristics (e.g., marital status, race/ethnicity, number of children). Using Fragile Families and Child Wellbeing (FFCW) data, Slack and colleagues [35] found that welfare-dependent families were more likely to depend on SNAP or Medicaid than on TANF or UI, regardless of employment status. In comparing these families’ use of benefit programs at the time their children were one year old vs. when their children were five years old, their findings (based on cluster-level program participation rates for eight programs) suggested that families might exhibit different patterns of multiple program use as family situations change.

Using Wisconsin’s state administrative data on WIC-receiving families, Yang and colleagues [36] found that low-income families with employed household heads were more highly dependent on Medicaid than on cash benefits. However, they did not account for the continuous sequences of multiple program use over time in their study. Rather, they only captured multiple program use at a single time point [36]. Although Slack and colleagues [35] clustered families by their employment status and eight single program participation statuses at two timepoints (i.e., child’s age one and child’s age five), their findings did not provide insights into how families bundled different program combinations or whether families remained enrolled in the same welfare benefit packages over time. Indeed, Slack and colleagues [35] only recorded the percentage of each cluster group receiving each benefit and did not examine how families enrolled in multiple benefits (i.e., more than two) simultaneously. By contrast, the present study of single mothers transitioning from employment to unemployment not only analyzed data from all 50 US states, but also intentionally focused on longitudinal patterns in multiple benefit program participation. This means that findings from our data are both more generalizable and more granular than those yielded by prior similar studies.

Employment status affects the family benefits packages accessed by low-income single mothers. Some programs (e.g., TANF) require work participation, while other programs (e.g., UI) require unemployment status. Further, research has shown that a change from employment to unemployment increased the likelihood of receiving a family benefit package, including UI, among low-income single mothers [17]. Thus, the months immediately following job loss are important transitional periods for studying access to cash benefits and how they are combined with in-kind supports. Two studies [37,38] measured employment instability in families who experienced at least one change from full-time to part-time or unemployment or from part-time employment to unemployment. However, neither study examined the relationships between program participation and employment status. The current study addresses this knowledge gap by examining changes in longitudinal patterns of multiple program participation after job losses, and by examining characteristics associated with multiple program participation sequences.

### Characteristics associated with multiple program participation and/or its sequence

Most studies examining the associations between participation in multiple programs and demographic, economic, and work-related characteristics have not focused on single-parent families. Nevertheless, findings on multiple program participation among families may be generally applicable to single-mother families and, consequently, are reviewed here. Prior research has found that multiple program recipients were more likely to be younger mothers [39]; less educated [10,40–42]; renters (rather than homeowners); and African American [10,39,41,42]. They were also more likely to have a work-limiting health condition [10], to be unemployed [10,40], or to live in a household with another adult who was unemployed or had a work-limiting health condition. The finding that multiple program recipients included a disproportionate number of younger mothers, people with socioeconomically disadvantaged conditions, and unemployed people emphasizes the importance of studying sequences of multiple program enrollment among single mothers after job loss.

Other characteristics, such as region, may also impact multiple program participation. For example, TANF-receiving families in urban areas were more likely to receive other benefits than their counterparts in suburban areas [43]. Likewise, family characteristics may predict participation in specific benefits programs. For example, a study using SIPP data found that, among low-income families, those with children were more likely to access both SNAP and Medicaid [44]. In addition, Hispanic mother families were found to have lower rates of multiple program participation than non-Hispanic families [39]. Citizenship status can also be a contributing factor to patterns of multiple program participation because citizenship status might determine program eligibility. SNAP serves a substantial number of households with citizen children and non-citizen parents [45]. Another study found that families that included non-citizens were more likely to use food programs and Medicaid than families in which all members were citizens [46]. Collectively, these studies indicate that individual circumstances and policy environments shape the complex experiences of multiple program participation among low-income single mothers.

Although the studies reviewed above provide some insights into multiple program participation, little is known about how low-income single mothers utilize combinations of programs following job losses, and the ways in which that use changes over time. Most studies of multiple program participation among low-income families have focused broadly on all household types (e.g., [32,39,47]), despite the fact that single-mother families face the highest risk of long-term unemployment and economic instability. To date, Chang and Wu’s [17] study is the only one to examine multiple program participation among single-mother families during the Great Recession. However, the methods used in that study could not provide insights into the sequences of multiple program participation and their heterogeneous clustering patterns. Further, the few studies of public program participation that have used sequence analysis or cluster analysis have focused on cash benefit programs [34] or changes in welfare packages [35] rather than on patterns of participation in various combinations of multiple programs after a job loss. Insights obtained from sequence and cluster analyses will improve our understanding of the timing and sequential patterns of complex multiple program participation.

The current study addresses the aforementioned gaps in the literature by analyzing how single mothers utilized welfare packages after transitioning from employment to unemployment during the Great Recession. By identifying (a) multiple program participation cluster groups among single mothers transitioning to unemployment and (b) characteristics associated with those cluster groups, our study seeks to generate fresh insights for interventions and policy reforms designed to improve the economic well-being of these vulnerable families in times of economic recession. Based on previous findings, we expected that many low-income single mothers would participate in multiple public benefits programs after transitioning from employment to unemployment. Particularly, we expected to find that single mothers continuously participated in in-kind social assistance programs (e.g., food and medical assistance) given their low-income status. Because UI benefits, by design, provided wage replacement for unemployed workers for up to 99 weeks during the Great Recession, we expect to see UI participation after job loss for qualified low-income single mothers. For those low-income single mothers who did not qualify for UI benefits, we expected to see TANF participation if they met the eligibility criteria and complied with the welfare-to-work rules (e.g., job search, attending training) set by their states of residence. We also expected to identify distinct patterns of use (i.e., clusters) of multiple benefit programs by single mothers, including a group of limited benefit access. Finally, we expected to identify individual and contextual characteristics that were associated with inclusion in each distinct cluster.

## Methods

### Data source and sample

This study drew on data from the first 15 waves of the 2008 SIPP panel, which were gathered at 4-month intervals from August 2008 to July 2013. This longitudinal, nationally representative survey of households across the United States includes detailed monthly information on sample characteristics, employment, earnings, family income, and government program participation. Most importantly for the current study, SIPP includes information about participation in a variety of social safety net programs, thus facilitating our exploration of patterns of multiple program participation of single mothers transitioning from employment to unemployment.

The sample for this study consists of single-mother household heads. To be included in our analysis, these single mothers had to: (1) be between 18–64 years old; (2) have at least one biologically related child under the age of 18 years in the household; (3) have a family income below 200% of the federal poverty line at study baseline; (4) complete interviews at all 15 waves; and (5) report at least one instance of two or more months of employment followed by two or more months of unemployment within the 60-month (i.e., 5-year) reporting period of the SIPP. The first three inclusion criteria resulted in an initial sample of 2,763 single mothers. Applying the fourth inclusion criteria yielded a sample of 870 single mothers. We tested the difference in characteristics between those with and without complete 60-month data and found no statistically significant difference in most of the baseline characteristics, except racial distribution and work disability. The sample with complete 60-month data (N = 870) had a lower proportion of black (14% vs. 31%) and a higher proportion of mothers with a work disability (18% vs. 6%) than those without complete 60-month data (N = 1,893). Additionally, because this model requires four consecutive months of data before and after a report of a change in employment status from employment to unemployment and the data spanned 60 months, reports made before the 4^th^ or after the 56^th^ month were excluded. Our exclusions resulted in a final analytic sample of 342 single mothers. We also tested for differences in characteristics between the 870 and 342 single mothers. We found no statistically significant differences in most baseline characteristics, except single mothers’ age, work disability, and homeownership, between the characteristics of participants included in the final study sample and those excluded from the sample, suggesting that attrition produced negligible bias in our analyses.

To construct our analytic dataset, we began each participant’s data point at the first transition from employment to unemployment and extended her data for 12 months after that transition point. The 342 mothers in our final sample were unemployed for an average of 5.2 months (SD = 3.5 months) and were employed for an average of 6.8 months (SD = 3.5 months) during the 12-month time period. According to research by Zedlewski and Nichols [48], more than half of low-income single mothers were unemployed for six months or more during a recession. Thus, we examined program participation during the 12 months after the transition (or for as many months as appeared in the data for those making the transition in the 49^th^ month or later). This approach allowed us to identify the salient program participation statuses that often preceded the transition from employment to unemployment. It also minimized the inclusion of rare sequences that could bias the cluster analysis conducted in Aim 3.

### Measures

#### Multiple program participation

We tracked monthly participation in TANF, SNAP, Medicaid (MA), and UI programs because these programs are designed to support low-income families or unemployed workers, and sizeable numbers of participants commonly used each of these benefits. A dichotomous variable (1 = received benefits; 0 = did not receive benefits) was used to record participation in each of the four programs for each month. Displaying one of the four single program participation statuses (TANF only, SNAP only, MA only, or UI only) indicated that a single-mother family participated only in that program in a given month (TANF only did not occur among our sample). In addition to single program participation statuses, we constructed multiple program participation statues. Displaying one of the multiple program participation statuses indicated simultaneous participation in two or more programs in a given month. The following statuses were identified: TANF and SNAP; SNAP and MA; SNAP and UI; MA and UI; TANF/SNAP/MA; SNAP/MA/UI; TANF/SNAP/MA/UI; and no participation in any program (No PP). (SNAP/TANF/UI is theoretically possible, but it did not occur among our sample.) Then, we used sequence analysis and cluster analysis of all observed program combination statuses to identify cluster groups of members sharing similar sequential patterns of program participation over the 12-month period. Our cluster analysis used a set of predetermined measures to maximize within-group homogeneity and between-group heterogeneity using methods detailed in the analytic approaches section.

#### Sociodemographic and economic characteristics

We analyzed how the following sociodemographic characteristics related to multiple program participation: mother’s age; marital status (never married, divorced, or widowed); education (high school degree and more, no high school); race/ethnicity (White [reference group], African American, Hispanic, and other); US citizenship (1 = yes, 0 = no); work-related disability (1 = yes, 0 = no); number of children; age of the youngest child; other adults in household (1 = yes, 0 = no); income-to-poverty ratio; homeownership (1 = homeowner, 0 = not a homeowner); residence in metro areas (1 = yes, 0 = no); residence in the southern region of the country (1 = yes, 0 = no); and unemployment rates in the participant’s state of residence at Wave 1. Given that our outcome variables (cluster groups of multiple program participation sequences) were derived from data over the entire study period, we used covariates measured at the baseline month instead of the time-varying covariates in our models. Summary statistics for single mothers’ sociodemographic and economic characteristics at baseline appear in Table 1.

**Table 1:** Sample characteristics of single mothers (*N* = 342).

Variable	Proportion/Mean	SD
**Age**	38.20	10.90
**Marital Status**		
** Never Married**	49.10%	
** Divorced/Widowed**	50.90%	
**High School**	78.10%	
**Race and Ethnicity**		
** White**	44.15%	
** African American**	32.85%	
** Hispanic/Other**	23.10%	
**US Citizen**	90.10%	
**Work Disability**	18.40%	
**Number of Children**	1.80	1.10
**Age of Youngest Child**	6.80	5.20
**Other Adult in Household**	21.9%	
**Income-to-Poverty Ratio**	111.52	101.73
**Homeownership**	27.50%	
**Residence in Metropolitan Area**	75.40%	
**Residence in South Region**	40.60%	
**State Unemployment Rate**	8.70	1.90

### Analytic approaches

For Aim 1 (examine longitudinal patterns of multiple program participation), the monthly program participation rates were summarized as a percentage of the sample of 342 mothers.

For Aim 2 (identify multiple program participation cluster groups), we first conducted sequence analysis [49] to identify patterns in multiple program participation over a 12-month period. We analyzed the sequences of the 11 program participation statuses [SNAP, SNAP/MA, SNAP/UI, SNAP/UI/MA, MA, TANF/SNAP, TANF/SNAP/MA, TANF/SNAP/UI/MA, UI, UI/MA, and no program participation (No PP)] over 12 months among 342 mothers. To examine the distance between pairs of sequences, optimal matching distances were computed using substitution costs based on transition rates observed in the data [50]. Thus, we were able to capture data on the order and duration of multiple program participation, improving our understanding of the timing and sequential patterns of complex multiple program participation. We visualized these sequential patterns using a sequence plot. The resulting optimal matching distance matrix calculated in Aim 2 was subjected to Ward hierarchical clustering, resulting in a 4-class solution. The optimal number of clusters (4) was determined using the squared multiple correlations, i.e., the proportion of variance explained by the clusters with values ≥ 70%, which is considered desirable (R-squared in this study was 75%).

We also examined the peaks in the Cubic Clustering Criterion plots, in which larger values indicate an optimal number of clusters and considered the number of observations in a cluster (≥ 10% of the data) that would allow meaningful analysis.

For Aim 3 (analyze characteristics associated with multiple program participation cluster groups), the clusters were compared to identify differences in demographic, family-level, and socioeconomic characteristics. To examine demographics and social characteristics associated with multiple program participation sequence clusters within the sample, we used the resulting clusters of the dependent variable in a multinomial regression model. The multinomial model is a type of regression model in which the log odds of the outcome are modeled as a linear combination of the independent variables. For covariate selection, we examined the relationship between the baseline demographics that were significantly associated with the cluster membership (i.e., mother’s age, marital status, race and ethnicity, work disability, income-to-poverty level, homeownership, residing in the southern region of the country, and state unemployment rate) from the bivariate analysis.

Based on the distribution of the different demographic variables and the limited sample size within categories, we selected mother’s age, marital status, race and ethnicity, work disability, income-to-poverty level, homeownership, residing in the southern region, and unemployment rate in the individual’s state for inclusion in the multivariate model. US citizenship was not included because 90% of the participants were US citizens. Data are presented as parameter estimates with their standard errors, as well as odds ratios and the likelihood ratio-based confidence intervals (also known as the profile-likelihood confidence intervals).

Sequence and cluster analyses were conducted using the sequence analysis process in the TraMineR package in R [51,52]. Sequence plots were plotted using the seqIplot function. Status distributions by month were plotted using the seqdplot function. The mean time spent in each sequence status was plotted using the seqmtplot function. The multinomial analysis was conducted using the Statistical Analysis Software version 9.4 (SAS Institute, Inc., n.d.).

## Results

### Single program participation rates

**Fig 1:**
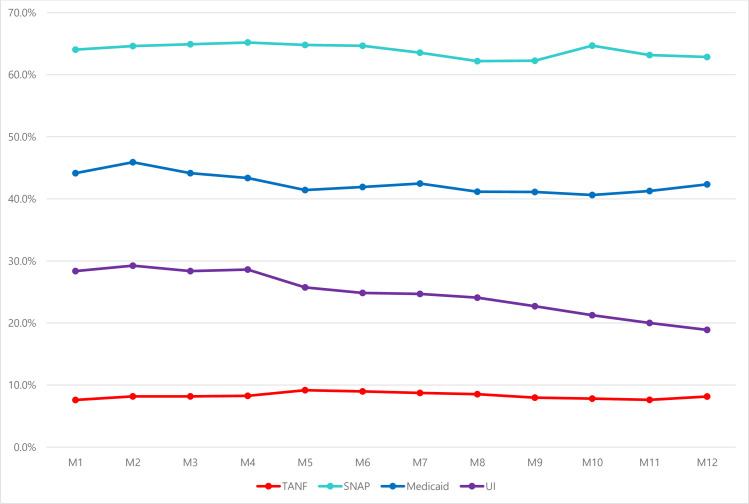
Single program participation rates after first transition from employment to unemployment. *Note*. SNAP = Supplemental Nutrition Assistance Program; TANF = Temporary Assistance for Needy Families; UI = Unemployment Insurance. M1-M12 represent the months after transitioning from employment to unemployment.

In the 12 months after single mothers in the study experienced their first transition from employment to unemployment, the likelihood of benefiting from one program remained generally steady. As shown in Fig 1, over the 12 months following a transition to unemployment SNAP participation hovered between 62% and 65% and Medicaid participation remained between 41% and 46%. The percentage of participants receiving UI declined over time. It was highest (about 29%) at the beginning of the first transition from employment to unemployment and fell to 19% by the 12^th^ month. TANF participation rates were consistently the lowest, remaining under 10%.

### Patterns of multiple program participation

**Fig 2:**
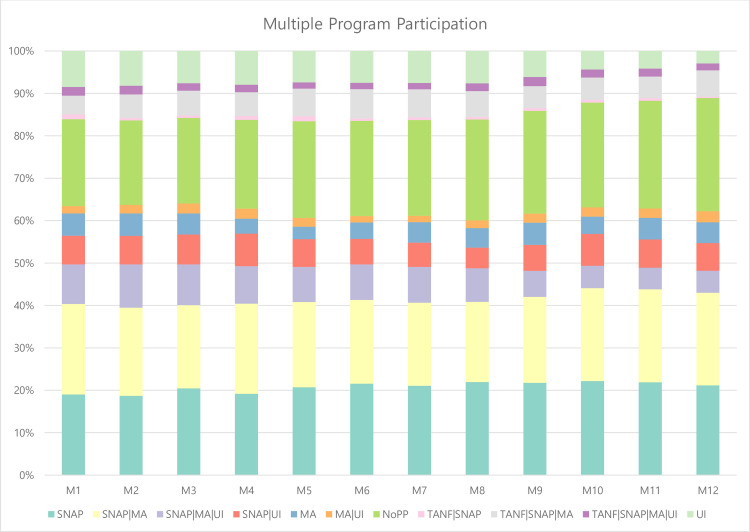
Patterns of multiple program participation after first transition from employment to unemployment. *Note*. MA = Medicaid; NoPP = No program participation; SNAP = Supplemental Nutrition Assistance Program; TANF = Temporary Assistance for Needy Families; UI = Unemployment Insurance. M1-M12 represent the months after transitioning from employment to unemployment.

The bars in Fig 2 show pattern results for the 11 different program combinations (including nonparticipants) of TANF, SNAP, MA, and UI observed in our study sample. For example, on average, SNAP only and SNAP/MA groups were tied for the second-largest group of multiple program participation. The size of SNAP only and SNAP/MA groups fluctuated slightly from about 19% to 21% of participants over the study period. Given that our data had very few TANF participants, it is unsurprising that multiple program participation rarely involved TANF. Similar to the drop observed in UI participation over the 12 months following unemployment from 29% to 19%, participation in SNAP/Medicaid/UI began at about 9.4% in the first month and fell to 4.7% by the 12^th^ month.

About 80% of single mothers were involved in various combinations of program participation in the 12 months after they experienced the first transition from employment to unemployment. However, nonparticipants comprised the largest of the groups in Fig 2. The proportion of nonparticipants increased over time from 20.5% in the first month to 24.0% in the 12^th^ month. That is, about 24% of single mothers were not receiving assistance from any of the programs studied 12 months after they transitioned to unemployment.

### Patterns and cluster groups of multiple program participation

**Fig 3:**
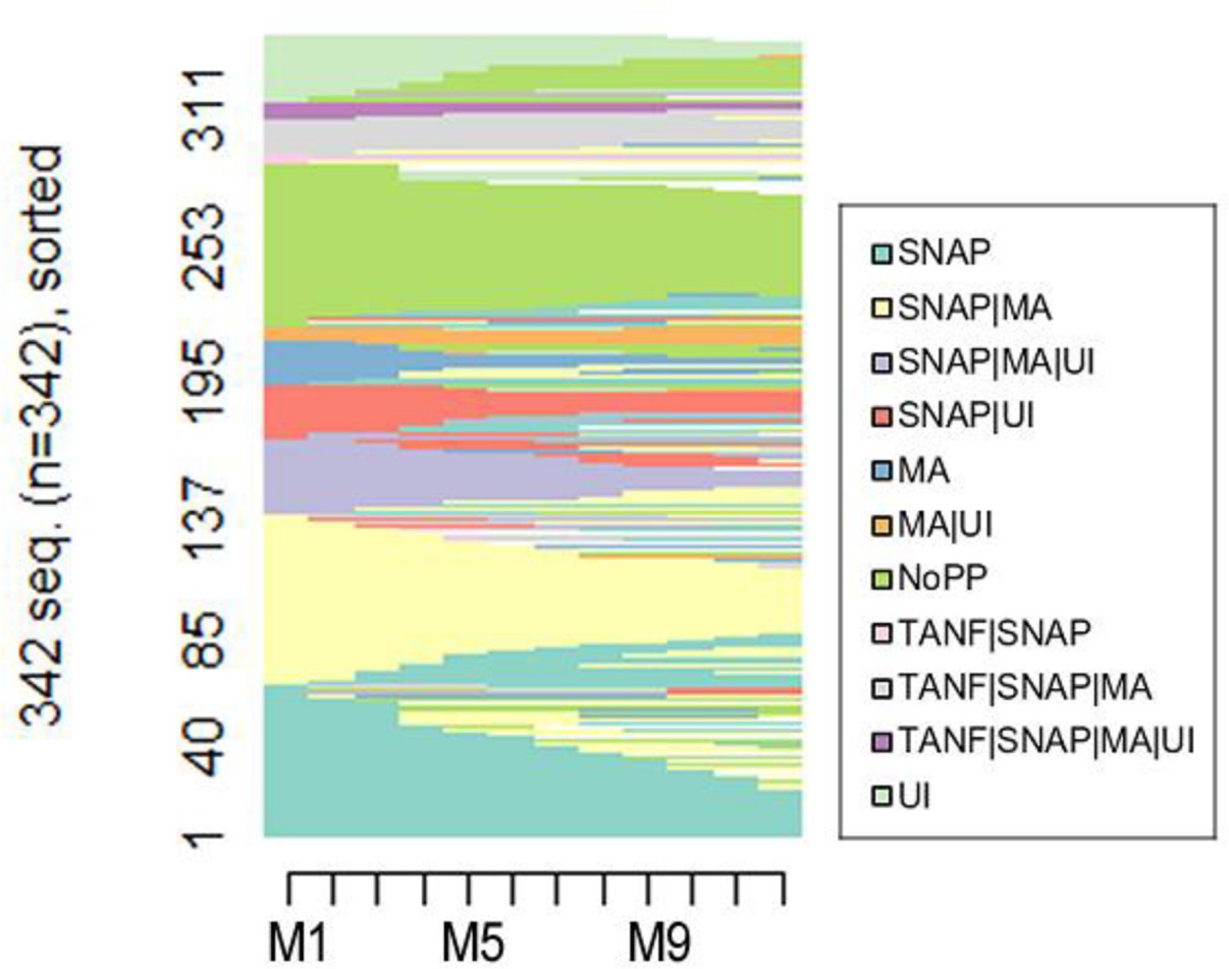
Sequence plot of multiple program participation. *Note*. MA = Medicaid; NoPP = No program participation; SNAP = Supplemental Nutrition Assistance Program; TANF = Temporary Assistance for Needy Families; UI = Unemployment Insurance. M1-M12 represent the months after transitioning from employment to unemployment.

Fig 3 displays the sequence plot of each individual’s transitions and sequences within and between the 11 statuses of TANF, SNAP, Medicaid, UI, and combinations thereof. Thus, it displays the timing and sequential pattern of complex multiple program participation of each single mother in the sample. Different colors indicate different combinations of program participation. A horizontal line represents the sequence of each individual in the sample, and the x-axis represents the passage of time, month by month, from the transition to unemployment. Some lines have many colors, indicating many transitions.

**Fig 4:**
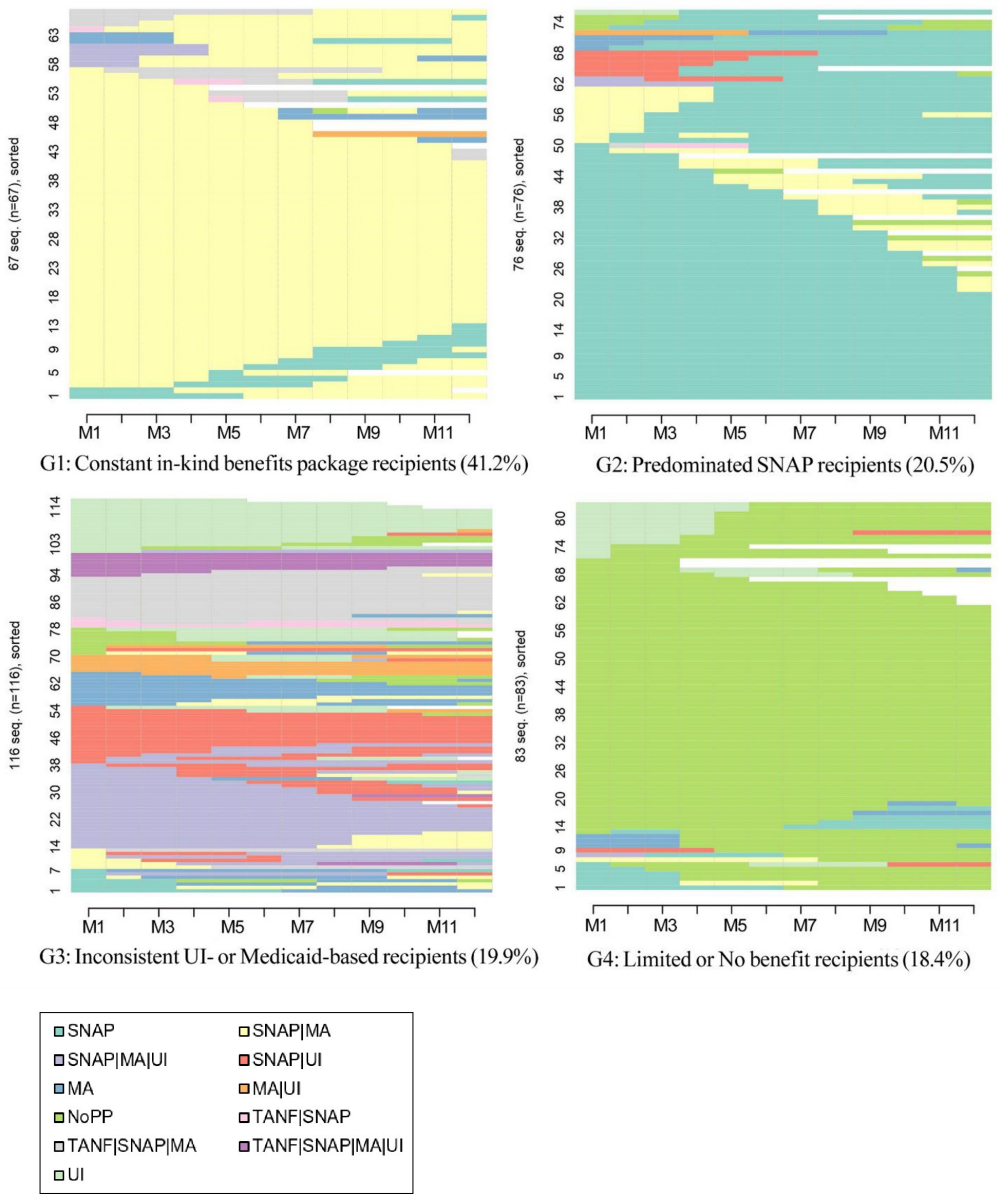
Sequence of multiple program participation by cluster groups. *Note*. MA = Medicaid; NoPP = No program participation; SNAP = Supplemental Nutrition Assistance Program; TANF = Temporary Assistance for Needy Families; UI = Unemployment Insurance. M1-M12 represent the months after transitioning from employment to unemployment.

Based on information about sequential patterns of program participation status, we identified clusters (i.e., plots) among the single mothers based on similarities and distance between every pair of sequences. A four-group solution maximized similarities within groups and differences between groups. The sequences of program participation of each group appear in Fig 4, which visually depicts the transitions from one combination of multiple programs to another. Here, too, horizontal lines represent an individual-level sequence of program participation over the 12 months after becoming unemployed.

Distinct patterns of program sequences determine an individual’s membership in a particular cluster. Fig 4 is a sequence index plot showing data for each of the four identified cluster groups. Each row reflects the multiple program participation sequence of one individual in the sample. It depicts both the heterogeneity of each cluster and between-cluster variation in multiple program participation over the focal 12 months.

The largest cluster was Constant In-Kind Benefits Package Recipients (Cluster 1). About 41.2% of participants received in-kind benefits (e.g., SNAP and MA) in every month of the study period, and a few received additional benefits for less than the entire 12-month period. Most mothers in this cluster continually received both SNAP and MA during the 12 months after the transition from employment to unemployment.

The second-largest cluster, representing 20.5% of the study sample, was Predominant SNAP Recipients, who received only SNAP for most of the study period. Most received only SNAP for the entire 12 months after they lost their job, but some also received MA along with SNAP for < 12 months. Some received SNAP and benefits from one of the other programs and received only SNAP after losing the other benefit; others added one of the other programs after a period of receiving only SNAP.

The third cluster, comprising 19.9% of the study sample, received benefits in an inconsistent pattern, which we call the Inconsistent UI- or MA-based Benefit Recipients. The variety of colors in Cluster 3 highlights this group’s inconsistent participation in benefits programs. Most families in this cluster experienced multiple transitions in program participation during the 12 months after job loss, although in most months they had UI or/and MA.

Lastly, approximately 18.4% of sampled single mothers were in the Limited or No Benefits Recipients cluster. Most single mothers in this group did not participate in any program in the 12 months after their transition from employment to unemployment, but some received a single benefit (e.g., SNAP or UI) or a combined benefit (e.g., SNAP plus UI plus Medicaid) for a limited time.

### Sociodemographic characteristics by multiple program participation cluster groups

The chi-square tests comparing sociodemographic characteristics of single mothers by each cluster group indicate several statistically significant differences across groups (see Table 2). Cluster 1, Constant In-Kind Benefits Package Recipients, was more likely to be Hispanic (29.1%) and more likely to not have citizenship (87.2%) than the other groups. Cluster 2, Predominant SNAP Recipients, were more likely than the other groups to be White (62.9%), to have higher rates of educational attainment (85.7% holding high school diplomas), to be homeowners (52.9%), to reside in the South (48.6%), and to have a higher income-to-poverty ratio. They were also less likely than all other groups to have a work-limiting disability (4.3%), and had the fewest average children (1.7 children) and the oldest average age (39.4 years old) among the four cluster groups. On average, the youngest child of families in Clusters 1 and 2 was older than the youngest child in families in Clusters 3 and 4. Cluster 3, the Inconsistent UI- or Medicaid-based Benefits Recipients, were slightly more likely to have a work-limiting disability (17.7%) and to live in states with slightly higher unemployment rates than all other groups. In contrast, single mothers in Cluster 4, Limited or No Benefits Recipients, were more likely to never have been married (68.2%), to be Black (42.9%), and to have a lower income-to-poverty ratio (63.9% PL) than all other groups. Additionally, they were less likely to have other adults in the household (12.7%), to live in an owner-occupied home (11.1%), and to reside in the South region. Their average age (32.9 years old) was the youngest among any of the groups.

**Table 2:** Summary statistics of cluster groups.

	Cluster 1: Constant in-kind benefits package recipients	Cluster 2: Predominant SNAP recipients	Cluster 3: Inconsistent UI- or Medicaid-based benefits recipients	Cluster 4: Limited or no benefits recipients	Chi-square significance
	(*N* = 141)	(*N* = 70)	(*N* = 68)	(*N* = 63)	
**Age**	36.0	39.4	36.2	32.9	*F* (3,338) = 11.24 *
**Marital Status**					*X*^2^ = 17.06 ***
** Never Married**	50.3%	32.9%	45.6%	68.2%	
** Divorced/Widowed**	49.7%	67.1%	54.4%	31.8%	
**High School**	74.5%	85.7%	73.5%	77.8%	*X*^2^ = 4.01
**Race and Ethnicity**					*X*^2^ = 24.69***
** White**	37.6%	62.9%	36.8%	46.0%	
** African American**	33.3%	14.3%	41.2%	42.9%	
** Hispanic/Other**	29.1%	22.9%	22.1%	11.1%	
**US Citizenship Status**	87.2%	90.0%	88.2%	98.4%	*X*^2^ = 6.42^+^
**Work Disability**	14.2%	4.3%	17.7%	17.5%	*X*^2^ = 7.01^+^
**Number of Children**	2.0	1.7	1.9	2.0	*F* (3,338) = 6.17
**Age of Youngest Child**	7.0	7.7	6.2	6.2	*F* (3,338) = 4.51
**Other Adult in Household**	23.4%	24.3%	25.0%	12.7%	*X*^2^ = 3.92
**Income-to-Poverty Ratio**	95.6	132.0	83.3	63.9	*F* (3,338) = 16.92***
**Homeownership**	19.9%	52.9%	25.0%	11.1%	*X*^2^ = 36.28***
**Residence in Metropolitan Area**	75.9%	70.0%	80.9%	76.2%	*X*^2^ = 2.24
**Residence in Southern Region**	42.6%	48.6%	44.1%	20.6%	*X*^2^ = 12.84***
**State Unemployment Rate**	5.6	5.8	5.9	5.8	*F* (4,337) = 5.59

^*+*^*p* < .10

*p < .05

**p < .01

***p < .001.

### Multinomial logistic model of multiple program participation cluster groups

Table 3 presents the multinomial logistic results for each cluster group compared with the Predominant SNAP Recipients group (Cluster 2), controlling for other covariates. Compared to White single mothers, single mothers who were African American were about four times as likely to be in-kind benefits recipients (OR = 4.201, 95% CI = [1.607, 10.982]); about six times more likely to be inconsistent UI- or Medicaid-based benefits recipients (OR = 6.219, 95% CI = [2.159, 17.916]); and about five times more likely to be limited benefits recipients (OR = 5.353, 95% CI = [1.848, 15.508]) than predominant SNAP recipients. Similarly, compared to those without a work disability, single mothers with a work disability were about five times more likely to be in Cluster 1 (Constant In-Kind Benefits; OR = 5.596, 95% CI = [1.558, 20.102]), seven times more likely to be in Cluster 3 (Inconsistent UI- or Medicaid-based Benefits; OR = 7.239, 95% CI = [1.885, 27.801]), and eight times more likely to be in Cluster 4 (Limited or No Benefits; OR = 8.336, 95% CI = [1.969, 35.294]) than in Cluster 2 (Predominant SNAP Recipients).

**Table 3:** Multinomial logistic model of multiple program participation cluster groups.

	Cluster 1 In-kind benefits recipients			Cluster 3 Inconsistent UI- or Medicaid-based benefits recipients			Cluster 4 Limited or no benefits recipients		
	coefficient	SE	odds ratio (95% CI)	coefficient	SE	odds ratio (95% CI)	coefficient	SE	odds ratio (95% CI)
**Age**	−0.020	0.019	0.980 (0.945, 1.017)	−0.027	0.021	0.974 (0.935, 1.014)	−0.028	0.022	0.973 (0.932–1.015)
**Marital Status (compared to never married)**									
** Divorced & Widowed**	−0.081	0.371	0.923 (0.445, 1.913)	0.284	0.448	1.329 (0.550, 3.208)	−0.419	0.469	0.658 (0.262, 1.653)
** African American**	1.435**	0.489	4.201 (1.607, 10.982)	1.828***	0.538	6.219 (2.159, 17.916)	1.678**	0.541	5.353 (1.848, 15.508)
** Hispanic/Other**	0.581	0.423	1.788 (0.779, 4.107)	0.433	0.505	1.542 (0.571, 4.161)	−0.660	0.577	0.517 (0.166, 1.609)
**Work Disability**	1.722**	0.650	5.596 (1.558, 20.102)	1.980**	0.684	7.239 (1.885, 27.801)	2.121**	0.734	8.336 (1.969, 35.294)
**Income-to-Poverty Ratio**	−0.002	0.002	0.998 (0.995, 1.002)	−0.005*	0.003	0.995 (0.990, 1.000)	−0.009**	0.003	0.991 (0.985, 0.998)
**Homeownership**	−1.191**	0.384	0.304 (0.143, 0.647)	−0.810	0.445	0.445 (0.185, 1.068)	−1.585**	0.535	0.205 (0.072, 0.587)
**Residence in South Region**	−0.708	0.367	0.493 (0.239, 1.014)	−0.804	0.416	0.448 (0.198, 1.014)	−1.946***	0.488	0.143 (0.055, 0.374)
**State Unemployment Rate**	−0.225	0.162	0.798 (0.580, 1.098)	0.046	0.196	1.047 (0.711, 1.540)	−0.093	0.201	0.911 (0.613, 1.353)
**Intercept**	3.114**	1.097		0.967	1.351		3.012**	1.279	

^*+*^*p* < .10

*p < .05

**p < .01

***p < .001.

Moreover, the income-to-poverty ratio was slightly lower in Cluster 3 (Inconsistent UI- or Medicaid-based Benefits; OR = 0.995, 95% CI = [0.990, 1.000]) and Cluster 4 (Limited or No Benefits; OR = 0.991, 95% CI = [0.985, 0.998]) than in Cluster 2 (Predominant SNAP Recipients). Mothers living in an owner-occupied home were more likely to receive only SNAP than other types of benefits (In-kind Benefits, OR = 0.304, 95% CI = [0.143, 0.647]; Inconsistent UI- or Medicaid-based Benefits, OR = 0.445, 95% CI = [0.185, 1.068]; or Limited or No Benefits (OR = 0.205, 95% CI = [0.072, 0.587]). Finally, compared to other single mothers, those living in the southern region were significantly less likely to be in the Limited or No Benefits cluster (OR = 0.143, 95% CI = [0.055, 0.374]) than in the Predominant SNAP Recipients cluster (Cluster 2).

## Discussion

Despite the well-documented economic vulnerability of single mothers in the United States [7,48,53,54], scant research on social safety net programs has focused on single mothers in poverty during or after periods of economic recession. A significant number of single mothers reduced work hours [55] or transitioned from employment to unemployment during and after the Great Recession [56]. The fact that public benefits programs have historically been implemented in ways that present barriers to participation for low-income single-mother families further underlines the urgent need for this study. Indeed, single-mother families remain underrepresented among social safety net program participants (e.g., [57,58]). Further, those who participate in these programs remain at increased risk for continued material hardship because the amount of support from these programs is insufficient to meet their basic needs [26,57–59]. To provide meaningful, effective initiatives for this higher-need population, it is necessary to understand the services and supports that are available to them after experiencing unemployment or underemployment and the factors impacting accessibility. The current study was designed to provide that information.

To this end, the current study analyzed the experiences of single mothers who became unemployed during the Great Recession. Using the SIPP panel, we selected a sample of 342 low-income single-mother families who were both employed and unemployed for at least 2 months within the 5-year SIPP panel period and analyzed their patterns of multiple program participation during the 12 months following their transition from employment to unemployment. Through this analysis, we identified four distinct cluster groups based on sequential patterns of multiple program participation over time, which we review below.

The largest cluster (41.2% of study participants) was the Constant In-Kind Benefits Cluster. Most people in this group consistently received food assistance and Medicaid over the 12 months after transitioning from employment to unemployment. In the second cluster, the Predominant SNAP Recipient Cluster (20.5% of study participants), most individuals only received SNAP during the 12 months after transitioning from employment to unemployment, although some did receive other benefits for a portion of this period. As its name implies, the Inconsistent UI- or Medicaid-Based Benefit Cluster (19.9% of study participants) did not show consistent program participation patterns during the 12 months after transitioning from employment to unemployment. Many families in this group received some type of support, often UI, MA, or SNAP, but experienced changes in program participation during the 12-month period after transitioning from employment to unemployment. Finally, in the Limited or No Benefits Cluster (18.4% of study participants), most individuals received no benefits, but a few received limited assistance for a short period during the 12 months after transitioning from employment to unemployment.

Our results revealed very limited participation in cash assistance programs overall. The Constant In-Kind Benefits Cluster (Cluster 1) and the Predominant SNAP Recipient Cluster (Cluster 2), which represent the use of in-kind benefits programs such as SNAP and Medicaid, showed relatively stable participation rates over time. By contrast, a large proportion of single mothers in the Inconsistent UI- or Medicaid-based Benefits Cluster (Cluster 3) had a sporadic pattern of participation in programs that involved a cash benefit receipt (UI or TANF). Even those who depended solely on UI benefits only received those benefits for a few months (i.e., the Limited or No Benefits Recipients, Cluster 4). The disproportionate share of African Americans and single mothers with work disability in Cluster 4 suggests that these groups encounter major obstacles when attempting to access social benefits. Additionally, our descriptive analysis indicated that only 11.4% of low-income single mothers ever received cash benefits during the 12 months after a job loss. Among those mothers, the average duration for the receipt of cash benefits was just 0.96 months (SD = 3.03 months) out of the 12-month period after becoming unemployed. Our results suggest that only a small portion of single mothers receive cash benefits, and those that do only receive them for a relatively short period of time after becoming unemployed.

Given that states’ stringent work sanctions and time limit policies have been associated with lower TANF participation among single mothers [60], the inconsistencies in cash benefit patterns may be attributable to state-level variations in the social safety net policies in the United States. Both the federal-state TANF and state UI programs vary considerably in fiscal capacity, program design, eligibility rules, benefit adequacy, and administrative procedures [12,61]. This lack of uniformity can create significant inconsistencies regarding eligibility, benefit amount, and participation duration, which may result in policies having disproportionate effects among some single mothers, depending on the state’s financial or political circumstances. For instance, some states have more generous and less restrictive programs to enable families to receive benefits longer than families in other states [62].

Other findings reveal varied consequences of uneven benefits provision in different parts of the country. After controlling for other individual characteristics and state unemployment rates, our multinomial logistic model revealed that, compared to those from other regions, single mothers in the South were less likely to be in the Limited or No Benefits Recipients group (Cluster 4) than in other groups during the first 12 months after transitioning into unemployment (Table in S1 Table). Our findings contradict suggestions in prior studies that southern states’ social safety net programs are more inaccessible and inadequate than those in other regions [63–65]. However, TANF programs in southern states tend to have stricter time limits, higher administrative burdens, and are less likely to offer categorical eligibility practice for multiple programs–all of which may limit low-income single-mother families’ consistent access to income support programs [64]. UI benefits in southern states also typically pay lower benefits to unemployed workers and to a smaller percentage of unemployed workers than UI benefits in other regions [63].

Because so few single mothers in the current data set actually received any cash benefits, the geographic differences in access to cash benefits likely had a limited impact on the results of the current study. Most of the states that declined to expand Medicaid under the Affordable Care Act were located in the South, meaning that these states have stricter eligibility criteria and less coverage compared to other states [66]. Because southern states have higher proportions of African Americans than other regions, these restrictions have also exacerbated racial and ethnic disparities in healthcare coverage [66] in this region and nationwide.

Regional or individual characteristics not considered in our model may explain the inconsistency between our findings and those of previous studies. For example, the racial distribution of poverty across regions and racial differences in unemployment duration might explain the discrepancies. There might be regional differences between the states, as variations in industrial infrastructure and human capital are the main factors that contribute to regional disparities in employment growth/economic performance [67]. Future studies should further explore how regional and state-level factors interact with individual characteristics to affect multiple program participation among low-income single mothers transitioning from employment to unemployment.

Our study’s findings should also be considered in light of several limitations. First, only 342 single mothers were included in our study because attrition reduced our sample size. Thus, findings may not be generalizable to the national low-income single mother population. Second, given that the data relied on self-reporting, there may have been instances of misreporting employment status or program participation, which would have introduced potential inaccuracies into the data. Third, we only analyzed data for single mothers in the 12 months following their transition from employment to unemployment. We did not address the impact of changes in economic and policy contexts on the sequence of participation patterns over the 5 years of data. These contexts include changes in the state level of employment, unemployment, and employment program utilization, as well as the impact of other income support programs. This confluence of potential factors impedes our ability to analyze the impact of long-term benefits of specific economic and policy contexts on single mothers. Finally, although our study retrospectively analyzed how single mothers utilized multiple social support programs in the 12 months after transitioning from employment to unemployment, we did not examine the potential impact of these programs on the hardships faced by single mothers. A logical next step for our research is to determine the impact of these varying patterns of program utilization on the economic hardships of single mothers transitioning from employment to unemployment.

There is an urgent need for policy interventions that address the vulnerabilities of low-income single-mother families by promoting their enrollment in social safety net programs. Strengthening the social safety net requires greater understandings of patterns of participation in welfare programs by single mothers, the factors that contribute to those patterns, and the impact of the varying combinations of social welfare programs on participants’ economic hardships. To this end, the current study brings to light (a) patterns and changes in multiple program participation among single mothers who experienced a transition from employment to unemployment during the Great Recession, and (b) the demographic characteristics associated with these varying patterns of participation. Further research is essential to determine the impact of these varying patterns of program utilization on the economic hardships of single mothers transitioning from employment to unemployment. Additionally, subsequent studies will explore associations between the patterns of multiple program use by single mothers and their efficacy in relieving financial hardships. The results of the current study can be used in conjunction with information learned in future research about the efficacy of patterns of multiple benefit use to inform changes in policy that improve access to the best combinations of safety net programs for single mother families facing economic hardship.

Although national trends show that single mothers are exceedingly vulnerable to employment and economic instability, welfare participation patterns of single mothers transitioning to unemployment during prolonged economic recessions had remained largely unexplored until now. Our study addresses this gap by examining how low-income single-mother families utilize different benefits, how these benefits or combinations of benefits vary over time, and the demographic factors associated with varying patterns of benefit utilization. This new knowledge is positioned to inform revisions to existing social welfare programs or the creation of new social welfare programs at an incipient moment in the United States’ economic recovery from the current recession so that these services will effectively meet the needs of low-income single mothers who become unemployed in the future.

## Supporting information

S1 Table Multinomial logistic model of multiple program participation cluster groups (PDF). (DOCX)
