# Supplementary material for: How do low-income single-mothers get by when unemployment strikes: Patterns of multiple program participation after transition from employment to unemployment
Source: PLoS One. 2022 Sep 22;17(9):e0274799. doi: 10.1371/journal.pone.0274799 (PMC9499290; doi:10.1371/journal.pone.0274799)
Supplement: S1 Table — (DOCX) [file pone.0274799.s001.docx]

**Supporting Information File**

The following supporting information is available for this article:

| **S1 Table. Multinomial Logistic Model of Multiple Program Participation Cluster Groups** | | | | | | | | | | | |
| --- | --- | --- | --- | --- | --- | --- | --- | --- | --- | --- | --- |
|  | **Cluster 1**  **In-kind benefits recipients** | | |  | **Cluster 2**  **Predominant SNAP recipients** | | |  | **Cluster 3**  **Inconsistent benefits recipients** | | |
|  | coefficient | SE. | odds ratio  (95% CI) |  | coefficient | SE. | odds ratio  (95% CI) |  | coefficient | SE. | odds ratio  (95% CI) |
| **Age** | 0.007 | 0.019 | 1.008  (0.969, 1.048) |  | 0.032 | 0.022 | 1.033  (0.990, 1.078) |  | 0.000 | 0.021 | 1.000  (0.959, 1.043) |
| **Marital status (compared to never married** |  |  |  |  |  |  |  |  |  |  |  |
| **Divorced/Widowed** | 0.417 | 0.423 | 1.517  (0.660, 3.489) |  | 0.525 | 0.469 | 1.690  (0.672, 4.252) |  | 0.746 | 0.478 | 2.109  (0.823, 5.404) |
| **Race (compared to white)** | | | | | | | | | | | |
| **African American** | −0.204 | 0.414 | 0.815  (0.361, 1.842) |  | −1.602** | 0.544 | 0.202  (0.069, 0.587) |  | 0.182 | 0.464 | 1.200  (0.482, 2.985) |
| **Hispanic/Other** | 1.289* | 0.500 | 3.630  (1.358, 9.701) |  | 0.674 | 0.568 | 1.961  (0.641, 5.998) |  | 1.128 | 0.575 | 3.091  (0.998, 9.574) |
| **Work Disability** | −0.239 | 0.532 | 0.788  (0.277, 2.243) |  | −2.081** | 0.726 | 0.125  (0.030, 0.520) |  | −0.034 | 0.536 | 0.966  (0.997, 2.773) |
| **Income-to-poverty Ratio** | 0.011** | 0.004 | 1.011  (1.004, 1.018) |  | 0.010* | 0.004 | 1.010  (1.002, 1.018) |  | 0.007 | 0.004 | 1.007  (0.999, 1.014) |
| **Homeownership** | 0.427 | 0.488 | 1.532  (0.587, 4.000) |  | 1.703** | 0.522 | 5.488  (1.964 15.335) |  | 0.782 | 0.553 | 2.185  (0.736, 6.486) |
| **Residence in South Region** | 1.187** | 0.435 | 3.276  (1.392, 7.710) |  | 1.790*** | 0.482 | 5.989  (2.323, 15.441) |  | 1.117* | 0.470 | 3.055  (1.213, 7.696) |
| **State unemployment rate** | −0.131 | 0.174 | 0.877  (0.623, 1.236) |  | 0.053 | 0.198 | 1.054  (0.714, 1.556) |  | 0.139 | 0.196 | 1.149  (0.781, 1.690) |
| **Intercept** | −0.014 | 1.066 |  |  | −2.786* | 1.272 |  |  | −2.123 | 1.316 |  |
| **p* < .05; ***p* < .01; *** *p* < .001  *Note*. In the multinomial model, cluster membership was the outcome with the cluster of ‘Limited or No Benefits Recipients’ (Cluster 4) used as a reference. | | | | | | | | | | | |
